# Radium and Its Medical Uses

**Published:** 1907-03-16

**Authors:** G. H. Graham

**Affiliations:** Medical Officer, Electrical Department, East London Hospital for Children


### RADIUM AND ITS MEDICAL USES.

Notes of a Lecture delivered at the Polyclinic on Tuesday, March 5,

By G. H. Graham, M.D., D.P.H., Medical Officer, Electrical Department, East London Hospital for Children.

Radium possesses therapeutic properties of considerable value. Its rarity in Nature and the difficulty of obtaining it make it a costly agent to employ; but those who have had the opportunity of working with it have obtained results which cannot be questioned. The rays given off from radium may be classified in three groups—namely, ($\alpha$), ($\beta$), and ($\gamma$) rays. For medical purposes the ($\alpha$) and ($\beta$) rays are the active agents. The ($\alpha$) rays are the only ones which possess bactericidal properties, though the ($\beta$) rays seemed to have the power of inhibiting the growth of micro-organisms.

Regarding its physiological effects, radium penetrates the skin, and is capable of producing sloughing. For therapeutic purposes radium is enclosed in small capsules of thin glass, or in a box having a mica window in the centre. Special celluloid films, metal discs, and pencils can also be obtained coated with radium. The dose of radium is regulated by several factors: (1) The radio-activity of the salt used; (2) the weight of the salt; (3) the density of the part treated; (4) the thickness of the capsule enclosing the radium; and (5) the duration of the exposure. No pain or sense of heat is felt during the application. Sometimes an appreciable effect can be recognised after the third exposure, but as a rule definite action occurs only in from seven to fourteen days. The problem is "What is taking place in the tissues during this latent period?" Probably there is constriction of the capillaries, and of other small vessels, followed by thrombosis. On the skin the action is an inflammatory one, with some phagocytosis. The skin is irritated, and vesication sometimes occurs, and may leave some pigmentation. If the exposure has been prolonged the skin may be destroyed, and the resulting ulcer may prove very difficult to heal. Following a moderately acute reaction a characteristic scar is formed, very white in the centre, and with a red marginal ring of dilated capillaries.

No authentic case of the successful curative action of radium on cancer has been reported. The various methods employed have produced no really stable results, apparently because they failed to attack the growth over the whole of its surface at the same time. In some cases complete cure of facial paralysis has been recorded under radium. The diminution and disappearance of lightning pains in tabetics and the return of sensation in anæsthetic patches in tabetics and lepers have also been recorded. Cases of chronic pruritis ani have been reported as cured after two sittings of ten minutes each with ten days' interval between, and details have been given of the entire relief of intense cutaneous hyperæsthesia following an attack of zona by applications of ten minutes' duration repeated in several places.

In dermatological practice radium, either alone or in combination with the Finsen treatment, has been used successfully in lupus vulgaris. It is especially useful in treating this disease in the nose, mouth, or other places where the light treatment cannot be applied, though when large areas are treated with radium the skin has been left less soft and pliable than when the Finsen treatment has been employed. In a case of lupus erythematosus, even where the Finsen treatment had only been partially effective, radium has produced a complete cicatrisation. In a case of Bazin's disease bromide of radium applied from fifteen to thirty minutes cleared up the subcutaneous nodules where central necrosis had not begun.

Capillary nævi are very amenable to applications of radium, the smaller ones quickly clearing up, but the larger ones requiring time. Polland has given details of an extensive cavernous angioma involving the right half of the face, which gradually contracted after long exposures to an active radium salt, and in the end left a healthy scar; the result, however, was not permanent, as three months later a fresh angioma formed at the edge of the scar. Paget's disease of the nipple has been shown by Hartigan as cured by the use of radium.

Warts, pigmented warts, and hairy moles, small sebaceous or dermoid cysts, such as are often seen in or adjacent to the nasal groove, and small fibrous thickenings of the skin are all speedily removed by radium. It has been used successfully in eczema and psoriasis. In these affections it is only necessary to give exposures of from one to five minutes' duration on each area, so that a fairly large surface can be treated in a reasonable time. Patches of chronic leucoplakia of the tongue have also been stated to disappear after two sittings. Secondary and tertiary syphilitic lesions have been successfully treated. A suitable method of treatment in skin affection is to cover the surface with cotton-wool made radio-active. Gelatine charged with radium emanations and spread on any suitable material acts perhaps better, as it retains the radio-activity for a longer period. On chancres it appears to have a beneficial influence. In ophthalmic practice trachoma has been successfully treated by radium. The action of radium in conjunctivitis, uveitis and central ulcer of the cornea has been favourably reported upon by different observers, as has also the relief of pain in rheumatic ophthalmia, chorditis, and orbital neuralgia.

In gynæcological practice cases of uterine fibroids accompanied by severe hæmorrhages have been treated by radium. After three or four exposures the bleeding and pain ceased and the fibroids lessened in size. Gonorrhœal urethritis, metritis, and extensive erosions and eversion of the cervix all rapidly improve with from two to four applications made with a glass tube containing a very highly active salt of radium.

